# Sorafenib for hepatocellular carcinoma patients beyond Milan criteria after orthotopic liver transplantation: a case control study

**DOI:** 10.1186/1477-7819-10-41

**Published:** 2012-02-17

**Authors:** Chieh-Lin Teng, Wen-Li Hwang, Yi-Ju Chen, Kuang-Hsi Chang, Shao-Bin Cheng

**Affiliations:** 1 Division of Hematology/Oncology, Department of Medicine, Taichung Veterans General Hospital, 160, Section 3, Chungkang Road, Taichung 407, Taiwan; 2 Department of Life Science, Tunghai University, 181, Section 3, Chungkang Road, Taichung 407, Taiwan; 3 Department of Medicine, Chung Shan Medical University, 110, Section 1, Jianguo N. Road, Taichung 402, Taiwan; 4 Division of General Surgery, Department of Surgery, Taichung Veterans General Hospital, 160, Section 3, Chungkang Road, Taichung 407, Taiwan; 5 Department of Research, Taichung Veterans General Hospital, 160, Section 3, Chungkang Road, Taichung 407, Taiwan

**Keywords:** HCC, OLT, Adjuvant therapy, Sorafenib

## Abstract

**Background:**

Orthotopic liver transplantation (OLT) is one of the most effective treatments for patients with hepatocellular carcinoma (HCC) within the Milan criteria. However, for patients beyond these criteria, the recurrence rate is higher and the prognosis is worse. Sorafenib is the only drug showing survival benefits in advanced HCC patients; however, its role in patients beyond the Milan criteria after OLT remains unclear and requires further investigation.

**Methods:**

As a case-control study, we retrospectively analyzed 17 Chinese patients beyond Milan criteria undergoing OLT for HCC. These patients were stratified into adjuvant (n = 5), palliative (n = 6), and control groups (n = 6).

**Results:**

Nine of 11 patients who received sorafenib after OLT needed dose reduction due to more than grade 2 side effects. The disease-free survival rates for patients with or without adjuvant sorafenib were 100% versus 37.5% (p = 0.034) at 6 months, 66.7% versus 9.4% (p = 0.026) at 12 months, and 66.7% versus 0.0% (p = 0.011) at 18 months, respectively. The overall survival rates for patients in palliative and control groups were 66.7% versus 40.0% (p = 0.248) at 6 months, 66.7% versus 40.0% (p = 0.248) at 12 months, and 50.0% versus 20.0% (p = 0.17) at 18 months, respectively. Patients in the adjuvant group had better overall survival rates than those in the palliative and control groups (p = 0.031) at 24-month follow-up.

**Conclusions:**

Adjuvant sorafenib could possibly extend both disease-free and overall survival for HCC patients beyond Milan criteria after OLT.

## Background

Hepatocellular carcinoma (HCC) is a highly prevalent malignancy, especially in Asia. Liver cirrhosis is the strongest predisposing factor for HCC, accounting for approximately 80% of patients with this disease [1]; therefore, the risk factors for liver cirrhosis are generally also the risk factors for HCC. In the United States, Europe, and Japan, hepatitis C virus (HCV) infection is the major etiology of liver cirrhosis and HCC [2]. Hepatitis virus B (HBV) infection, however, is the leading cause of HCC development in most Asian countries other than Japan [3]. In addition to HBV and HCV infection, alcoholic cirrhosis and metabolic disorders can also act as risk factors for HCC. With an increased understanding of epidemiology and tumor biology of HCC, better surveillance strategies for high-risk patients have been proposed, including the use of serum alpha-fetoprotein and abdominal ultrasound at intervals of 6 months [4]. The goal of this surveillance program is to detect HCC in the early stage so that curative interventions can be introduced. Unfortunately, the prognosis of patients with HCC remains dismal because early HCC can be detected in only 30% of cases [5]. Thus, developing effective but tolerable therapeutic modalities for advanced HCC is an important and urgent issue.

The current management of HCC is essentially based on the Barcelona Clinic Liver Cancer (BCLC) staging system. Briefly, for patients with very early (stage 0) and early-stage diseases (stage A), surgical resection, percutaneous ethanol injection, radiofrequency ablation (RFA), and orthotopic liver transplantation (OLT) are the therapeutic options with intention to cure [5]. A high incidence of recurrence, which cannot be prevented after resection and RFA, is the major obstacle for these treatments. However, OLT differs from local resection and RFA, and has the potential to provide oncological and cirrhotic liver clearance. A study by Mazzaferro et al. [6] shows that the overall survival (OS) at 5 years can be approximately 70% in patients fulfilling Milan criteria. For patients with advanced or unresectable disease, however, treatment options, such as transarterial chemoembolisation (TACE) and sorafenib, are only palliative [7]. Although being far from a curative drug, sorafenib remains one of the most encouraging successes among the treatments of HCC in the past decade.

Sorafenib is a multiple tyrosine kinases inhibitor. Raf, vascular endothelium growth factor, platelet-derived growth factor, and c-kit are its target molecules. It is believed that clinical benefits from sorafenib are because it has both anti-proliferative and anti-angiogenic effects [8], as shown by the SHARP study, wherein sorafenib was demonstrated to increase the median OS from 7.9 to 10.7 months in advanced HCC patients with Child-Pugh A cirrhosis [9].

Although a retrospective study conducted by Fan et al. [10] shows that the long-term relapse-free survival for HCC patients beyond Milan criteria receiving OLT is 61.5%, the role of OLT in the treatment of large or multifocal HCC remains controversial. A strategy for prolonging both disease-free and OS in patients in this scenario is a challenging but critical issue. The role of sorafenib in such patients remains unclear and needs further investigation. For this purpose, we have conducted a case-control study and retrospectively reviewed 17 Chinese HCC patients who were beyond the Milan criteria but underwent OLT in our institution, 11 of whom received sorafenib in either adjuvant or palliative settings. This study aimed to evaluate the survival benefits provided by sorafenib in these patients.

## Methods

### Patients

As a case-control study, 17 consecutive Chinese patients with diagnoses of HCC confirmed histologically undergoing OLT were enrolled in this study retrospectively from December 2004 to February 2011. All the 17 patients were beyond the Milan criteria. Milan criteria was defined as having a single tumor ≤ 5 cm or up to 3 separate lesions with none larger than 3 cm, no evidence of gross vascular invasion, and no regional nodal or distant metastases when they underwent OLT [6]. Our patients comprised 13 males and 4 females, with a median age of 55 years (range, 39-79 years). Patients were stratified into adjuvant (n = 5), palliative (n = 6), and control groups (n = 6). Patients in the adjuvant group were those who received adjuvant sorafenib therapy within 6 weeks after OLT until further disease progression. Patients in the palliative group were those who received palliative sorafenib therapy when local or distant recurrence occurred after OLT. Sorafenib would not be discontinued for patients in palliative group until unacceptable toxicity or patients' death. Patients in the control group were those who received neither adjuvant nor palliative sorafenib. Patients in each group had previously received radiotherapy, TACE, chemotherapy, or RFA before or after OLT when active lesions were present. The average number of days for follow up was 438.3 (range, 67-1714 days). This project was approved by the institution's review board (Taichung Veterans General Hospital, Taiwan).

### Tumor staging classification, surveillance, and survival analyses

According to the performance scale, tumor status, and liver function, we used the BCLC [7] system for tumor staging in our study. Following OLT, all patients underwent chest X-ray, abdominal computed tomography, and serum alpha-fetoprotein assessment as regular surveillance at intervals of 3 months. The treatment response was evaluated according to the RECIST criteria [11]. Local recurrence and distant metastases as detected by imaging were used to confirm disease progression. Disease-free survival (DFS), which was defined as the period between the day of OLT and the day of local or distant recurrence, was evaluated to clarify whether adjuvant sorafenib therapy was beneficial. The OS in this study was defined as the period from OLT or HCC recurrence to patients' death for any reasons.

### Immunosuppressants for rejection prevention and treatment

For the 11 patients who underwent OLT, except case 7 who was administered sirolimus (4 mg/M^2 ^per day) for rejection prevention, the other 10 patients received tacrolimus (0.1 mg/kg per day). Rejection episodes were treated by methylprednisolone at an initial dose of 10 mg/kg per day, and adjusted according to the clinical response.

### Statistical analysis

One-way ANOVA and chi-square tests were used for the comparison of patient characteristics in each group. We calculated the OS and DFS using the Kaplan-Meier method. Statistical significance was set at *p *< 0.05. All the statistical analyses were performed using SPSS software, version 11.5 (SPSS Inc., Chicago, IL, USA).

## Results

### Patient characteristics

All the clinical characteristics of the enrolled patients are summarized in Table 1. Briefly, all the HCC cases in our cohort were HBV or HCV related, accounting for 64.7% (11/17) and 35.3% (6/11), respectively. Liver cirrhosis stratification revealed that 58.8% (10/17), 23.5% (4/17), and 17.6% (3/17) of cases to be Child-Pugh A, B, and C categories. According to the BCLC staging system, only 1 patient was classified as stage A, 6 patients at stage B, 7 patients as stage C, and 3 patients as stage D before transplantation. Most patients had received various treatments before OLT, including TACE in 11, RFA in 4, and surgery in 2 patients. Except two patients who had living liver donors, the other 15 patients received their livers from cadaveric donors. The average time of sorafenib use for patients in adjuvant and palliative groups was 284 (range: 6 to 630) and 291 (range: 59 to 544) days, respectively. Comparisons of clinical characteristics among the patients from the 3 groups are listed in Table 2.

**Table 1:** Characteristics of 17 patients with hepatocellular carcinoma undergoing liver transplantation

**Pt No**.	Pt Group	Sex	Age	Viral Hepatitis	Liver cirrhosis Child-Pugh	Pre-OLT BCLC	Histology	AFP (ng/mL)	Pre-OLT Treatment	Donor Type	Relapse Location	Post Relapse Treatment	Sorafenib Dose (mg)	Current Status
1	Adjuvant	M	50	HBV	A	B	N/A	N/A	TACE	CD	Liver	TACE, R/T	400	PD
2	Adjuvant	M	50	HBV	A	A	Mod. Diff.	3.29	OP, RFA	CD	Nil	Nil	600	CR
3	Adjuvant	M	55	HBV	A	B	N/A	3115	Nil	CD	Nil	Nil	400	CR
4	Adjuvant	M	61	HCV	A	B	Mod. Diff.	153	TACE, RFA	CD	Nil	Nil	400	CR
5	Adjuvant	M	51	HBV	B	B	P. Diff.	25.4	TACE	CD	Nil	Nil	400	CR
6	Palliative	M	56	HBV	C	D	N/A	12.31	TACE	CD	Potal vein	Nil	400	PD
7	Palliative	M	48	HBV	A	C	N/A	5567	TACE, RFA	CD	Liver	TACE, R/T	800	Dead
8	Palliative	M	51	HBV	B	C	P. Diff.	22.12	TACE	LD	Lung	C/T	400	CR
9	Palliative	M	48	HBV	A	B	P. Diff.	5.07	Nil	CD	Liver	R/T, RFA	800	Dead
10	Palliative	F	61	HCV	B	D	N/A	7745	TACE, RFA	CD	Lung	Nil	200	Dead
11	Palliative	M	55	HBV	C	D	N/A	N/A	Nil	CD	Portal vein	R/T	400	Dead
12	Control	F	78	HCV	A	B	N/A	N/A	TACE	CD	Lung	Nil	Nil	Dead
13	Control	M	39	HBV	A	C	P. Diff.	24.21	TACE	CD	Liver	TACE, OP, C/T	Nil	Dead
14	Control	M	57	HBV	B	C	P. Diff.	6.38	TACE	CD	Liver	OP	Nil	Dead
15	Control	F	62	HCV	A	C	N/A	9058	OP, RFA	CD	Liver	TACE	Nil	Dead
16	Control	M	62	HCV	A	C	Mod. Diff.	13.74	TACE	CD	Nil	Nil	Nil	Dead
17	Control	F	62	HCV	C	C	P. Diff.	98589	Nil	LD	Lung	Nil	Nil	Dead

Pt: patient, M: male, F: female, HBV: hepatitis B virus, HCV: hepatitis C virus, OLT: orthopedic liver transplant, BCLC: Barcelona Clinic Liver Cancer, N/A: not available, Mod. Diff.: moderately differentiated, P. Diff.: poorly differentiated, AFP: alpha-fetoprotein, N/A: not available, TACE: transarterial chemoembolisation, OP: operation, RFA: radiofrequency ablation, CD: cadaveric donor, RFA: radiofrequency ablation, CD: cadaveric donor, LD: living donor, R/T: radiotherapy, C/T: chemotherapy, PD: progressive disease, CR: complete remission, RFA: radiofrequency ablation, CD: cadaveric donor, LD: living donor, R/T: radiotherapy, C/T: chemotherapy, PD: progressive disease, CR: complete remission

**Table 2:** Comparisons of clinical characteristics among different groups

		Total n = 17 (%)	Adjuvant Group n = 5 (%)	Palliative Group n = 6 (%)	Control Group n = 6 (%)	P value
**Age (Mean **± **SD)**		55.6 ± 8.6	53.4 ± 4.7	60.0 ± 12.5	53.2 ± 5.1	0.324
**Sex**						0.133
F		4 (23.5)	0 (0)	1 (16.7)	3 (50.0)	
M		13 (76.5)	5 (100.0)	5 (83.3)	3 (50.0)	
**Viral Hepatitis**						0.135
HBV		11 (64.7)	4 (80.0)	5 (83.3)	2 (33.3)	
HCV		6 (35.3)	1 (20.0)	1 (16.7)	4 (66.7)	
**C-P Classification**						0.522
A		10 (58.8)	4 (80.0)	2 (33.3)	4 (66.7)	
B		4 (23.5)	1 (20.0)	2 (33.3)	1 (16.7)	
C		3 (17.6)	0 (0)	2 (33.3)	1 (16.7)	
**Pre-OLT BCLC Staging**						0.011
A		1 (5.9)	1 (20.0)	0 (0)	0 (0)	
B		6 (35.3)	4 (80.0)	1 (16.7)	1 (16.7)	
C		7 (41.2)	0 (0)	2 (33.3)	5 (83.3)	
D		3 (17.6)	0 (0)	3 (50.0)	0 (0.0)	
**Donor Type**						0.624
CD		15 (88.2)	5 (100)	5 (83.3)	5 (83.3)	
LD		2 (11.8)	0 (0)	1 (16.7)	1 (16.7)	
**1^st ^Relapse Site**						0.052
Liver		6 (35.3)	1 (20.0)	2 (33.3)	3 (50.0)	
Lung		4 (23.5)	0 (0)	2 (33.3)	2 (33.3)	
Portal vein		2 (11.8)	0 (0)	2 (33.3)	0 (0.0)	
No relapse		5 (29.4)	4 (80.0)	0 (0)	1 (16.7)	

SD: standard deviation, F: female, M: male, HBV: hepatitis B virus, HCV: hepatitis C virus, C-P: Child-Pugh, OLT: orthotopic liver transplantation, BCLC: Barcelona Clinic Liver Cancer, CD: cadaveric donor, LD: living donor

### Sorafenib dose adjustment and toxicity

The targeted daily dose of sorafenib was 800 mg. The dose of sorafenib was adjusted by patients' tolerability and safety, according to the National Cancer Institute Common Terminology Criteria version 3.0. Dose reduction was required if the adverse effects were greater than grade 2. Nine of 11 patients (81.8%) needed dose reduction in four weeks after initiation of sorafenib in our cohort. Only one patient discontinued sorafenib due to intolerable adverse effect (grade 4 fatigue). The average dose in our study was 472.7 mg per day with a range of 200-800 mg.

### Adjuvant sorafenib improved DFS in HCC patients who received OLT

**Figure 1:**
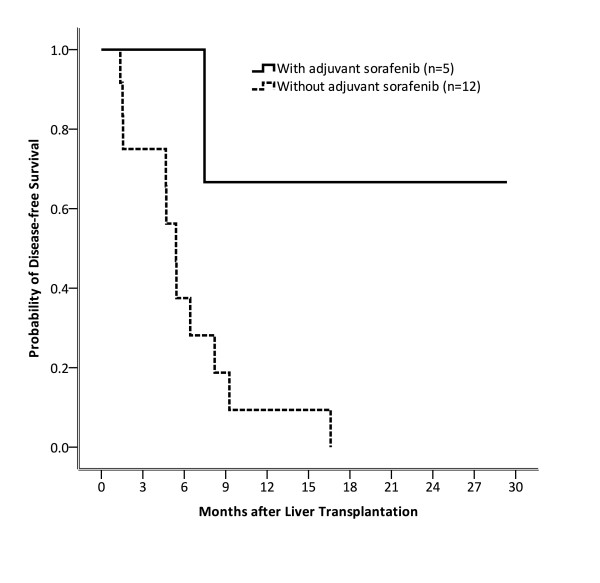
**The disease-free survival rate was analyzed by the Kaplan-Meier method**. In patients with or without adjuvant sorafenib, it was 100% versus 37.5% (p = 0.034) at 6 months, 66.7% versus 9.4% (p = 0.026) at 12 months, and 66.7% versus 0.0% (p = 0.011) at 18 months, respectively. Adjuvant sorafenib could significantly improve disease-free survival in patients beyond the Milan criteria after orthopedic liver transplantation.

In our cohort, 5 of 17 patients received adjuvant sorafenib, while 12 did not. The results of this analysis are shown in Figure 1. Briefly, the DFS rates for patients with or without adjuvant sorafenib were 100% versus 37.5% (p = 0.034) at 6 months, 66.7% versus 9.4% (p = 0.026) at 12 months, and 66.7% versus 0.0% (p = 0.011) at 18 months, respectively. Adjuvant sorafenib therapy thus significantly improved DFS in patients beyond the Milan criteria after OLT.

### Palliative sorafenib could possibly provide survival benefits after HCC recurrence

**Figure 2:**
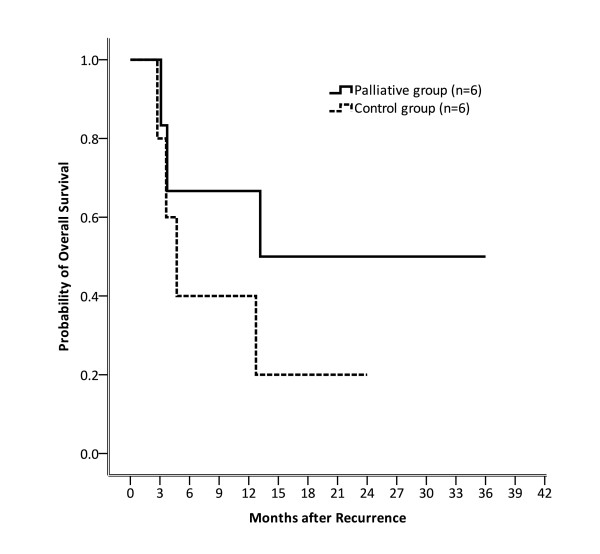
**Overall survival rates for patients in the palliative and control groups were 66.7% versus 40.0% (p = 0.248) at 6 months, 66.7% versus 40.0% (p = 0.248) at 12 months, and 50.0% versus 20.0% (p = 0.17) at 18 months, respectively**. There were no statistical differences between these two groups.

Subsequently, we evaluated whether palliative sorafenib provided survival benefits in HCC patients with recurrent disease. Because all the patients in the palliative group showed active disease progression, the progression-free survival was not calculated. Results for OS are shown in Figure 2. The OS rates for patients in the palliative and control groups were 66.7% versus 40.0% (p = 0.248) at 6 months, 66.7% versus 40.0% (p = 0.248) at 12 months, and 50.0% versus 20.0% (p = 0.17) at 18 months, respectively. Except case 16 who died of HCV reactivation, all the other mortalities in both the palliative and control groups occurred due to HCC progression. Although there was no statistical significance, patients receiving palliative sorafenib tended toward superior OS according to the Kaplan-Meier survival curve.

### Adjuvant sorafenib improved OS

**Figure 3:**
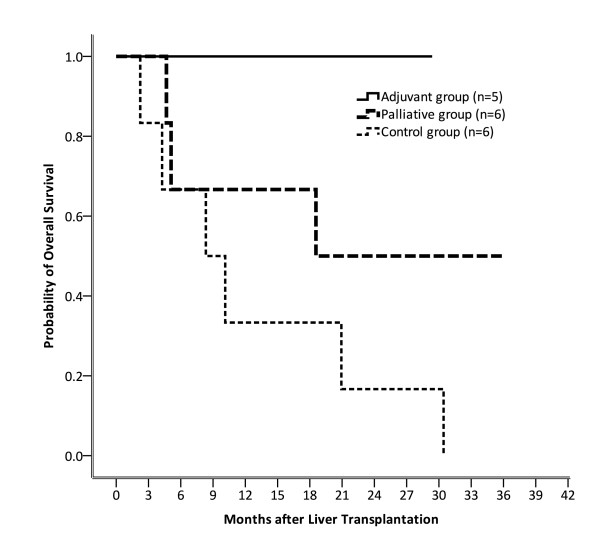
**At 24-month follow up, the overall survival rates for patients in adjuvant, palliative, and control group were 100% (5/5), 50% (3/6), and 16.7% (1/6), respectively**. Patients in the adjuvant group had better overall survival than those in the palliative and control groups (p = 0.031).

We further evaluated whether sorafenib could improve OS for HCC patients after OLT. The results of this evaluation are shown in Figure 3. All the patients in the adjuvant group (n = 5) were alive at 12, 18, and 24 months after OLT. For patients in the palliative group, 66.7% (4/6) patients were alive at 12 and 18 months. Further, 50% (3/5) patients were alive at 24 months. For patients in the control group, however, 33.3% (2/6) were alive at 12 and 18 months. Only 1 patient in this group remained alive at 24 months after OLT. Thus, patients in the adjuvant group showed a better OS rate than those in the palliative and control groups (p = 0.031) at 24-month follow up. Considering these results together, we assumed that better DFS by adjuvant sorafenib therapy may contribute to better OS for HCC patients beyond the Milan criteria after OLT.

## Discussion

The Milan criteria are considered a universal standard for the selection of HCC patients for OLT. The debate regarding the feasibility of transplanting a patient beyond the Milan criteria has not been resolved thus far. Although the relapse rate is higher, OLT remains the only possible curative treatment for these patients. The prevention of disease recurrence is extremely important in these patients. Zhang et al. [12] evaluated the efficacy of post-OLT adjuvant chemotherapy with FOLFOX regimen to patients beyond the Milan criteria, and concluded that adjuvant FOLFOX could not prevent tumor recurrence but may improve the survival. One of the major concerns for this study, however, was the possibility that the cytotoxic agents used could damage the transplanted livers, rendering sorafenib as a potential solution for reducing the possibility of tumor recurrence. However, there are only a few studies that have examined this issue. Similarly, a phase 3 placebo-controlled randomized trial is currently ongoing for evaluating whether sorafenib can be an effective adjuvant therapy for HCC patients after tumor resection or local ablation (STORM trial). The results of this study would be available in the near future, which can possibly establish the rationale of adjuvant sorafenib therapy after OLT in HCC patients beyond the Milan criteria.

Although prospective and randomized-control studies regarding the role of adjuvant sorafenib on post-OLT HCC patients beyond Milan criteria remain unavailable, there is a retrospective study conducted by Saab et al. [13] suggesting that sorafenib has the potential benefit to extend both DSF and OS in high-risk HCC patients after OLT. In this study, the DFS and OS at 1 year is 85.7% and 87.5%, respectively. Although there is no significant statistical analysis in Saab's cohort, this study provides initial but important evidence that adjuvant sorafenib could be effective for patients receiving OLT for HCC for better DFS and OS. Results from our study further demonstrated that patients receiving adjuvant sorafenib had significantly better DFS than those who did not. Our preliminary data showed that DFS rates in patients with or without adjuvant sorafenib were 100% and 37.5% (p = 0.034) at 6 months, 66.7% and 9.4% (p = 0.026) at 12 months, and 66.7% and 0.0% (p = 0.011) at 18 months, respectively. To our knowledge, this is the first report showing statistical significance for survival benefits in post OLT HCC patients who are beyond Milan criteria.

The second aim of our study was to investigate the role of palliative sorafenib in post-OLT patients with relapsed HCC. One of the first studies regarding this issue was conducted by Kim et al. [14], involving 9 patients who received sorafenib after OLT for HCC recurrence. Survival benefits were not discussed in this study because the purpose of the study was to evaluate drug safety and feasibility, and 6 of the 9 patients required dose reduction due to side effects. A study conducted by Yoon et al. [15] further surveyed the survival benefits by sorafenib in this setting. Thirteen patients within the Milan criteria were retrospectively reviewed, showing that the median progression-free survival and OS was 2.9 months and 5.4 months, respectively. Yoon et al. therefore suggested that sorafenib could be a feasible treatment for recurrence HCC in patients with OLT. In our study, because all the patients in the palliative group had their disease in progression at the first follow up after initiating the treatment, progression-free survival could not be obtained in our analyses. Although no statistical significance was observed, a trend toward superior OS was noted for the patients in this group as compared to those in the control group. Variations in tumor recurrence location in the palliative and control groups could possibly explain why OS did not reach statistical significance in this analysis.

It has been demonstrated that TACE can prolong survival in patients with unresectable HCC [16]. In our cohort, only 33.3% (2/6) patients in the palliative group experienced recurrence over liver parenchyma, indicating that TACE could not have improved survival in the remaining 4 patients. In the control group, however, except 1 patient who died of HCV reactivation, recurrence over the liver occurred in 3 of 5 patients (60%). All of these 3 patients had received TACE for their liver recurrence. This variation suggested that a larger portion of the patients in the control group could possibly benefit from TACE. Our speculation can be partially supported by a study conducted by Tan et al. [17], who demonstrated that in patients with HCC recurrence after OLT, the median OS for patients undergoing TACE with sorafenib and TACE alone was 14 and 6 months, respectively (p = 0.005). Patients who receive TACE with sorafenib thus had a better median OS than those who received sorafenib alone. Performing TACE in patients with recurrent HCC after OLT would thus further enhance the treatment efficacy of palliative sorafenib therapy.

Was superior DFS by adjuvant sorafenib able to result in superior OS? Our results showed that the answer to this question was positive. Our data showed that patients in the adjuvant group had better OS than those in the palliative and control groups at 24-month follow up (p = 0.031). This result supported the study by Saab et al., that adjuvant sorafenib could significantly improve both DFS and OS in post-OLT HCC patients beyond the Milan criteria.

However, the retrospective nature and the small size of our cohort were the major limitations of our study. In addition, all patients in the adjuvant group were at BCLC stage A or B, while most of patients in the palliative and control groups were at C and D (p = 0.011). These findings suggested that the low recurrence rate in the adjuvant group could be partially because of the better BCLC stage, instead of sorafenib alone. Further, a heterogeneous dose of sorafenib raised a new issue: the most optimal dose, either in the adjuvant or palliative setting, for HCC patients after OLT needs to be examined.

In summary, our preliminary data showed that adjuvant sorafenib improved both DFS and OS in HCC patients beyond the Milan criteria after OLT. Palliative sorafenib could possibly prolong OS in post-OLT patients with disease progression. Performing TACE in these patients for liver recurrence might further provide survival benefits. Prospective and randomized control studies with larger cohorts are urgently warranted to answer these questions.

## Abbreviations

**BCLC: **Barcelona Clinic Liver Cancer; **DFS: **disease free survival; **HBV: **hepatitis B virus; **HCC: **hepatocellular carcinoma; **HCV: **hepatitis C virus; **OLT: **orthopedic liver transplantation; **OS: **overall survival; **RFA: **radiofrequency ablation; **TACE: **transarterial chemoembolisation.

## Conflict of interests

Dr. Shao-Bin Cheng received lecture fee from Bayer Pharmaceutical Co., Ltd.. Other authors declare that they have no conflict of interest.

## Authors' contributions

Chieh-Lin Teng participated in the writing of the paper and research design.

Wen-Li Hwang participated in the performance of the research.

Yi-Ju Chen participated in the performance of the research.

Kuang-Hsi Chang participated in data analysis.

Shao-Bin Cheng (corrsponding author) participated in the writing of the paper and research design.

All authors read and approved the final manuscript.
